# A Novel Surgical Treatment Approach for Vertical Root Fractures of Endodontically Treated Molars: A Report of 3 Cases

**DOI:** 10.3390/jcm14248966

**Published:** 2025-12-18

**Authors:** Nuo Chen, Chang Lu, Xinling He, Yuexing Zheng, Ying Yang, Wei Fan

**Affiliations:** The State Key Laboratory of Oral & Maxillofacial Reconstruction and Regeneration, Key Laboratory of Oral Biomedicine Ministry of Education, Hubei Key Laboratory of Stomatology, School & Hospital of Stomatology, Wuhan University, #237 Luoyu Road, Wuhan 430079, China; 2023283040024@whu.edu.cn (N.C.); m17359293969@163.com (C.L.); hexinling114@whu.edu.cn (X.H.); zyx1529755@163.com (Y.Z.)

**Keywords:** vertical root fracture, molar, intentional replantation, resin, bioceramic cement

## Abstract

**Background:** Vertical root fracture (VRF) is a severe complication of endodontically treated teeth with a poor prognosis. Despite many tentative tooth-preserving approaches, the current main treatment remains tooth extraction or root resection, which is largely due to the difficulty in balancing the mechanical strength for fracture fixation and biological properties for periodontal healing. Moreover, all documented reports regarding VRF repairing so far were limited to anterior teeth and premolars. Thus, the objective of this case report was to present a novel surgical treatment approach for repairing VRF of molars. **Methods:** Three patients (2 females, 1 male; aged 30–33 years) with endodontically treated molars (Tooth #46, #16, #37) diagnosed with VRF were treated with a dual-layered repair approach with modified fracture lines and retention forms through intentional replantation. **Results:** After 18, 21, and 36 months of follow-up, respectively, all three cases showed no clinical symptoms, normal tooth mobility and periodontal probing, as well as reduced periradicular radiolucency on radiographs. Root resorption or ankylosis was not observed. **Conclusions:** The novel surgical treatment approach demonstrates effectiveness in preserving endodontically treated molars with VRF, but its long-term treatment results for various VRF of molars need further randomized and controlled clinical investigations.

## 1. Introduction

Vertical root fracture (VRF), which is defined as a complete or incomplete longitudinal crack extending along the long axis of the root [1], is a serious complication with a poor prognosis in endodontically treated teeth [2]. Although tooth extraction is usually the most favored and predictable treatment for teeth with VRF [3], various tooth-preserving strategies have also been described in many case reports, including the root resection [4], intracanal medication [5], CO_2_ laser treatment [6,7], fracture re-cementation with adhesive resins [8,9] or sealing with bioceramic materials such as MTA [10] and biodentine [11]. Although the root resection could help preserve the multiple-rooted tooth, such as molars with VRF, by removing the affected root, the stress load and distribution on the operated tooth during the mastication function would be unfavorable for the rest root(s), thus increasing the fracture risk of the rest root(s). Considering this problem, many tentative methods have been reported for preserving the root with VRF through repairing the VRF [12,13].

Despite documented successes of various VRF repairing methods within different follow-up periods, a critical challenge persists in the selection of fracture repairing materials, i.e., materials that can effectively balance the mechanical strength for fracture fixation and biological properties for periodontal healing. A resin+iRoot BP Plus dual-layered repairing approach has been reported for the VRF of endodontically treated incisors and premolars, which showed a successful result in 12–24 months follow-up periods [14,15]. This approach tried to solve the inherent limitations of single-material repairing treatment, i.e., adhesive resins could provide mechanical strength for fracture fixation but have limited bioactivity for periodontal healing (thus prone to recurrent periodontal pockets) [16]; in contrast, bioceramic cements such as iRoot BP Plus demonstrate excellent bioactivity for periodontal tissue regeneration [17], yet lack sufficient mechanical bonding strength to stabilize fractures [18].

Furthermore, current reports about VRF repairing treatment are limited to anterior teeth and premolars. The lack of reports on VRF repairing treatment of molars is probably due to the difficulty in treatment and the higher mastication stresses undergone by molars, which is prone to induce fracture recurrence after repair. Additionally, molars exhibit uneven stress distribution due to their multi-rooted structure, further complicating repair durability. It has been reported that the modification of the fracture lines with retention forms in combination with adhesive resin could increase the fixation strength for fractures of the incisor and premolar [14,15]; however, due to these anatomical and functional differences, whether this method is also effective for VRF of molars remains unknown.

Based on these concerns, the present case series reported for the first time that three endodontically treated molars with VRF were successfully managed with a novel resin+iRoot BP Plus dual-layered repairing approach in combination with retention-form modification of fractures.

## 2. Materials and Methods

### 2.1. Clinical Techniques

**Figure 1:**
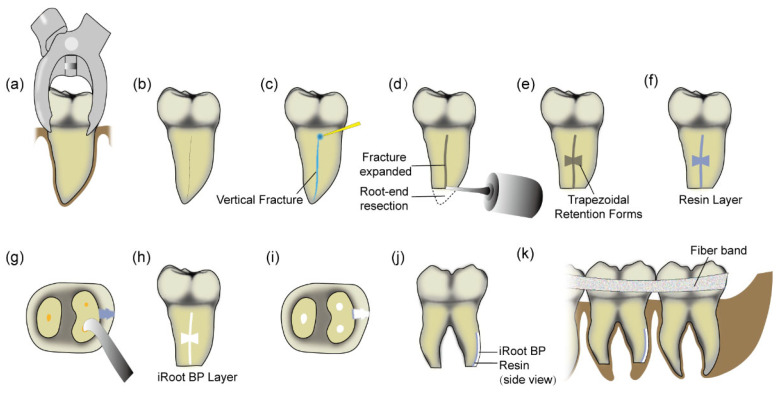
Schematic illustration of surgical procedures. (**a**) Tooth extraction. (**b**) Original vertical root fracture line. (**c**) Methylene blue staining. (**d**) Fracture expansion and 3 mm apical resection. (**e**) Preparation of trapezoidal retention forms. (**f**) Filling of the fracture and retention forms with resin. (**g**) Retrograde preparation. (**h**) Coverage of the resin surface with iRoot BP Plus. (**i**) Retrograde filling. (**j**) Side view of dual-layer filling. (**k**) Replantation and fixation.

The entire process was performed under aseptic conditions by the same experienced endodontist following the general protocol described below:

(1) Preparation and Tooth Extraction (Figure 1a): Local anesthesia was administered to patients using a 4% articaine solution containing 1:100,000 epinephrine (Zorcaine, Acteon Pharma, Mérignac, France). The use of an elevator during extraction was avoided. The supra-alveolar fibers were circumferentially dissected using a probe, and the affected tooth was extracted atraumatically with conventional extraction forceps. The beaks of the forceps were firmly positioned on the crown above the cementoenamel junction. To gradually loosen the tooth, a slow and controlled buccolingual luxation force was applied [19]. Then, the tooth was wrapped in saline-saturated gauze to maintain continuous hydration, while the blood-filled socket was protected with sterile gauze to minimize contamination of the socket [20,21].
(2) Extra-oral Repairing (Figure 1b–j): All subsequent extra-oral procedures were completed within 15 min under a dental microscope (Zumax Medical Co., Ltd., Suzhou, China). During the extraoral procedure, the tooth was handled exclusively by its crown using a gauze pad moistened with sterile normal saline, avoiding contact with the periodontal membrane area of the affected tooth. A saline irrigation syringe was used to continuously instill normal sterile saline onto the root surface. First, the granulation tissue on the root surface was meticulously debrided with a curette, after which the root surface was stained with methylene blue to visualize the vertical fracture line. A high-speed handpiece equipped with a fissure bur and cooled with sterile saline was used to expand the fracture line along its course to approximately 1 mm in width and 1.5–2 mm in depth. To enhance fixation stability, two trapezoidal retention forms were prepared at the middle and on both sides of the fracture line, with an external width of 3 mm, an internal width of 2 mm, and a depth of 1.5–2 mm. In the meantime, the apical 3 mm of the root was resected, and a 3 mm retrograde cavity was prepared using a 28 kHz ultrasonic tip (#7). Following application of the bonding agent, light-curing adhesive resin (3M ESPE, St Paul, MN, USA) was filled into the fracture line and retention forms. Upon full curing, approximately 0.5–1 mm of the surface resin was removed. Both the remaining resin surface and the apical root canal were obturated with iRoot BP Plus cement (Innovative Bioceramix Inc., Vancouver, BC, Canada).
(3) Replantation and Stabilization (Figure 1k): The repaired tooth was cleaned with saline and replaced into the socket with gentle pressure. After replantation, occlusal adjustment was performed to avoid any potential occlusal interference. The tooth was then splinted to adjacent teeth with glass fiber splints (Kuraray, Shanghai, China) for 1 month to ensure stabilization.

An immediate postoperative radiograph (Kavo Focus, Tuusula, Uusima, Finland) was taken to confirm accurate root resection, proper material placement, and correct tooth repositioning. The patient was also provided with detailed postoperative oral hygiene instructions.

### 2.2. Follow-Up

Splints were removed 1 month postoperatively. Clinical and radiographic follow-up visits were scheduled every 6 months or at the patients’ convenience. Clinical evaluation focused on the presence of symptoms, gingival swelling, sinus tracts, periodontal probing, tooth mobility, and recovery of masticatory function. Radiographic evaluation assessed the reduction in periradicular radiolucency and the occurrence of root resorption or ankylosis.

Treatment success was defined as the absence of clinical symptoms, no sinus tract and periodontal inflammation, periodontal probing depth ≤ 4 mm, normal tooth mobility and masticatory function, and the absence or reduction in periradicular radiolucency. Treatment failure was defined as the persistence of clinical symptoms, sinus tract, periodontal probing depth >4 mm, high tooth mobility, unchanged or enlarged periradicular radiolucency, and occurrence of root resorption or ankylosis [20].

### 2.3. Case Reports

These cases were conducted in accordance with the Declaration of Helsinki (2024) and received approval from the Ethics Committee of the School & Hospital of Stomatology. All patients were informed about the treatment plan, benefits, and potential complications. Written informed consent was obtained from each patient.

**Figure 2:**
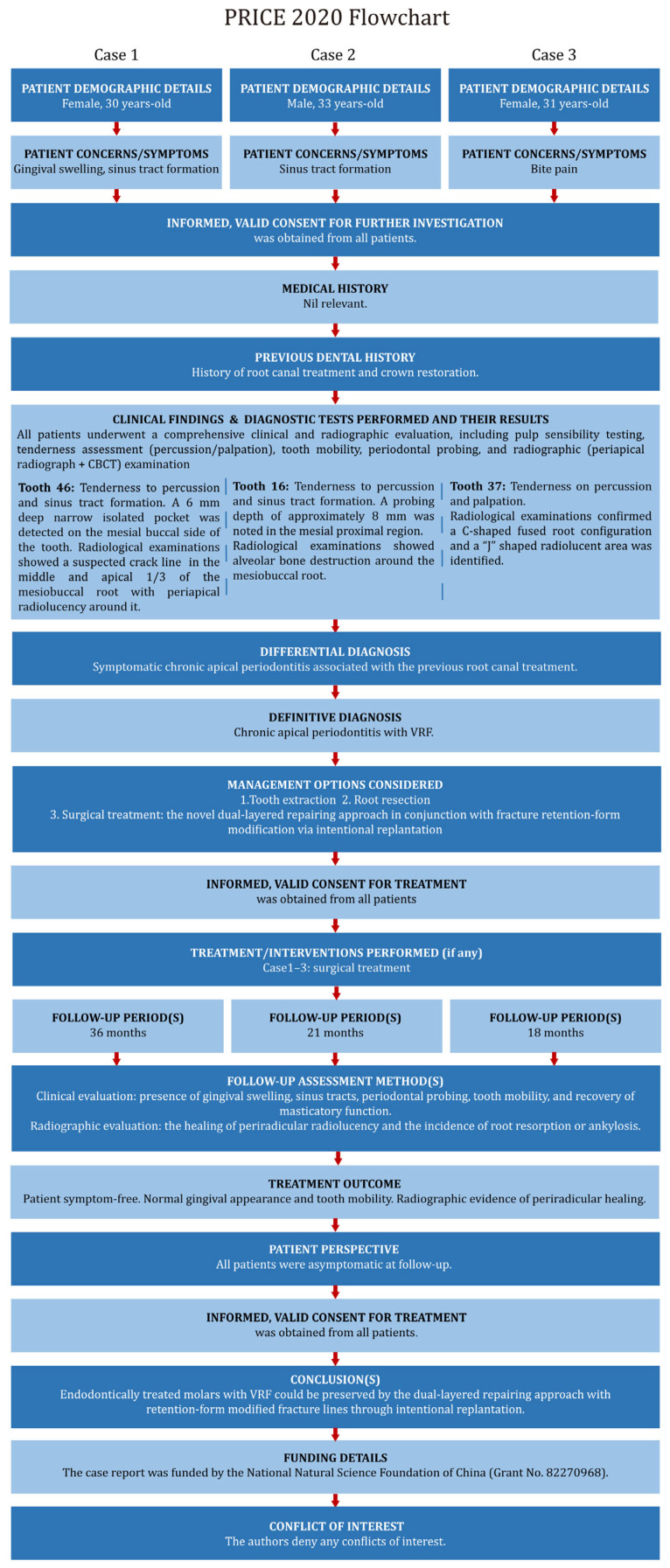
PRICE 2020 flowcharts.

This case report has been written according to Preferred Reporting Items for Case reports in Endodontics (PRICE) 2020 guidelines (Figure 2) [22].

For intraoral picture recording, a Nikon Z7ii Camera (Nikon, Tokyo, Japan) was used. All periapical radiographs were acquired using the paralleling technique and the VistaNet/VistaScan Nano Easy system (Carestream Health Inc., Rochester, NY, USA). Initial CBCT scans were performed using a CBCT machine (J. Morita MFG, CORP, Kyoto, Japan) with the following parameters: 90-kV peak, 7.0 mA, 60 × 60 mm field-of-view, and a 0.125-mm^2^ voxel size.

#### 2.3.1. Case 1

**Figure 3:**
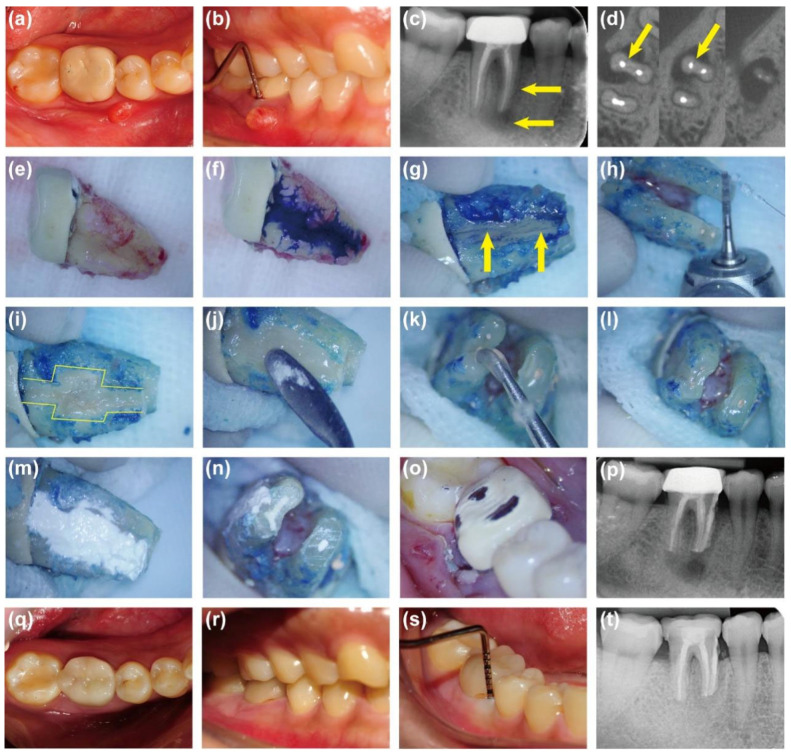
Photographs and radiographic images of case 1. (**a**,**b**) Pre-operative intraoral photographs showing sinus tract and deep periodontal probing depth. (**c**) Pre-operative radiograph showing periradicular rediolucency (arrows). (**d**) Pre-operative CBCT: A suspected fracture line (arrows) was observed in the middle and apical thirds of the mesio-buccal root. (**e**) Extraction of Tooth #46. (**f**) Methylene blue staining. (**g**) A vertical root fracture line (arrows) identified after staining. (**h**) Excision of the 3 mm root apex. (**i**) Fracture expansion and preparation of trapezoidal retention forms. (**j**) Filling of the fracture and retention forms with resin. (**k**,**l**) Retrograde preparation. (**m**) Coverage of the resin surface with iRoot BP Plus. (**n**) Retrograde filling. (**o**) Replantation. (**p**) Immediate post-operative radiograph. (**q**–**s**) Intraoral photographs at 36 months showing normal periodontal appearance and probing depth. (**t**) Periapical radiograph showing significant reduction in periradicular radiolucency at 36 months.

A 30-year-old female visited our department with a chief complaint of gingival swelling and sinus tract formation on the right posterior mandibular region for six months. She reported a history of root canal treatment and crown restoration on the mandibular right first molar (FDI tooth No. 46) six years ago. Clinical examination revealed that tooth #46 was restored and tender to percussion. A 6 mm deep, narrow, isolated pocket was detected on the mesial buccal side of the tooth, with sinus tract formation in the corresponding area (Figure 3a,b). Pre-operative radiograph and CBCT images confirmed a large radiolucent area around the buccal side of the mesial root. Additionally, a suspected crack line was noted in the middle and apical 1/3 of the mesiobuccal root (Figure 3c,d). Based on the examinations, tooth #46 was diagnosed as VRF. As described above, the surgical treatment through intentional replantation was performed on tooth #46 (Figure 3e–p). The last follow-up review at 36 months demonstrated that tooth #46 remained asymptomatic with normal gingival appearance, 4 mm periodontal probing depth, and normal tooth mobility (Figure 3q–s). Radiographic examination showed a significant reduction in periradicular radiolucency (Figure 3t). The patient was satisfied with the treatment outcome.

#### 2.3.2. Case 2

**Figure 4:**
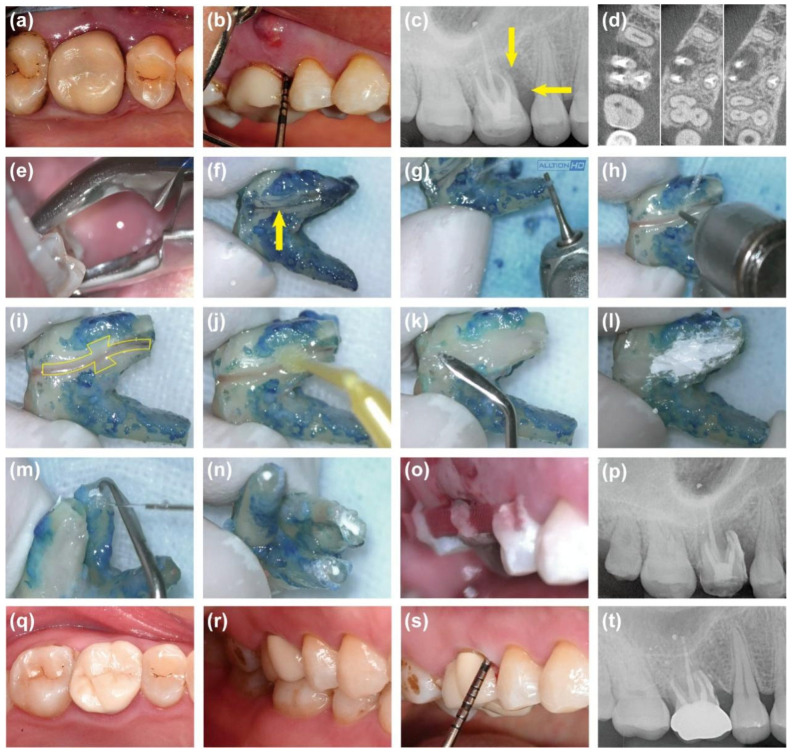
Photographs and radiographic images of case 2. (**a**,**b**) Pre-operative intraoral photographs showing sinus tract and deep periodontal probing depth. (**c**) Pre-operative radiograph showing the radiolucent area (arrows). (**d**) Pre-operative CBCT. (**e**) Extraction of Tooth #16. (**f**) Methylene blue dying showing fracture line (arrow). (**g**) Excision of the 3 mm root apex. (**h**) Fracture expanding. (**i**) Preparation of trapezoidal retention forms on both sides of the fracture. (**j**) Application of bonding agent. (**k**) Filling of the fracture and retention forms with resin. (**l**) Coverage of the resin surface with iRoot BP Plus. (**m**) Retrograde preparation. (**n**) Retrograde filling. (**o**) Replantation. (**p**) Immediate post-operative radiograph. (**q**–**s**) Intraoral photographs at 21 months showing normal periodontal appearance and probing depth. (**t**) Periapical radiograph at 21 months showing periradicular healing.

A 33-year-old male patient was referred due to the development of a draining sinus affecting the maxillary right first molar (FDI tooth No. 16) over 8 months. He reported that tooth #16 had undergone root canal treatment and full crown restoration 2 years prior. Clinical examination of tooth #16 revealed an intact ceramic crown, along with tenderness to percussion and palpation. A probing depth of approximately 8 mm was noted in the mesial proximal region, and a sinus tract was identified on the buccal gingiva (Figure 4a,b). Radiological examinations for tooth #16 revealed that adequate obturation of all root canals, accompanied by alveolar bone destruction around the mesiobuccal root (Figure 4c,d). Given the clinical and radiographic findings, tooth #16 was suspected of VRF, and the patient underwent the surgery as described above (Figure 4e–p). A 21-month follow-up evaluation confirmed a successful treatment outcome, evidenced by the patient’s asymptomatic status, normal periodontal appearance, and 4 mm probing depth, normal tooth mobility, and radiographic evidence of periradicular healing (Figure 4q–t).

#### 2.3.3. Case 3

**Figure 5:**
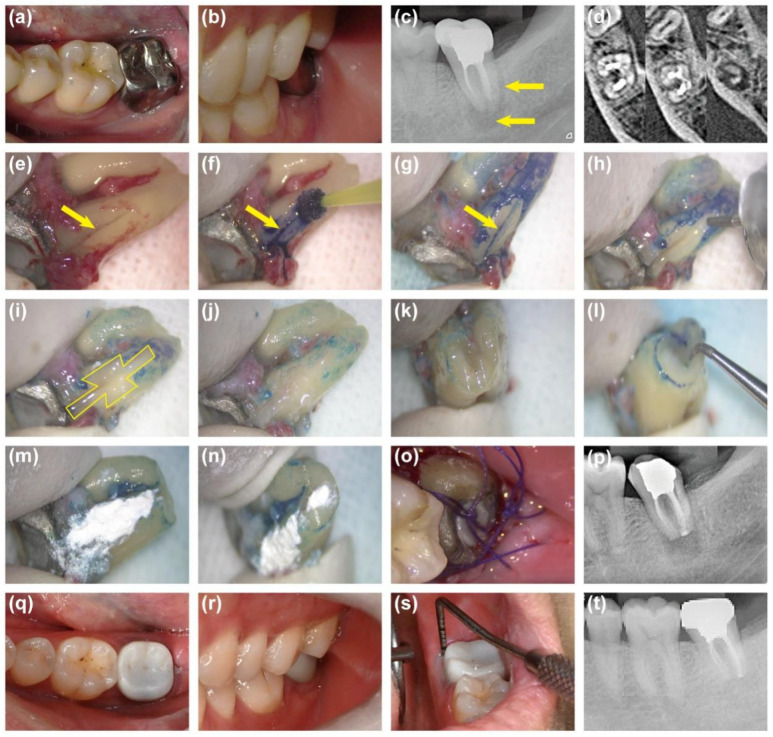
Photographs and radiographic images of case 3. (**a**,**b**) Pre-operative intraoral photographs. (**c**) Pre-operative radiograph showing periradicular J-shaped radiolucency (arrows). (**d**) Pre-operative CBCT. (**e**) Extraction of Tooth #37. (**f**) Methylene blue staining. (**g**) A vertical root fracture line identified after staining (arrow). (**h**) Fracture expansion. (**i**) Preparation of trapezoidal retention forms. (**j**) Filling of the fracture and retention forms with resin. (**k**) Excision of the 3 mm apex. (**l**) Retrograde preparation. (**m**) Coverage of the resin surface with iRoot BP Plus. (**n**) Retrograde filling. (**o**) Replantation. (**p**) Immediate post-operative radiograph. (**q**–**s**) Intraoral photographs at 18 months showing normal periodontal appearance and probing depth. (**t**) Periapical radiograph at 18 months showing periradicular healing.

A 31-year-old female presented to our department with a chief complaint of pain while chewing and biting on her mandibular left second molar (FDI tooth No. 37) over the past month, which had previously received RCT and a full crown restoration in another hospital. Clinical examination of tooth #37 revealed a full metal crown restoration and tenderness on percussion and palpation on the buccal gingival mucosa, with no sinus tract, and a deep periodontal pocket was detected (Figure 5a,b). Radiological examinations for tooth #37 confirmed root canal obturation and a C-shaped fused root configuration. A “J” shaped radiolucent area was identified at the distal aspect as well as the apex of the fused root (Figure 5c,d). The patient opted for surgical treatment as previously described (Figure 5e–p). During the surgical process, a VRF was identified on the distolingual surface of the root (Figure 5e–g). At the 18-month follow-up, the patient exhibited satisfactory clinical outcomes without symptoms, with normal periodontal appearance and 3–4 mm probing depth. Radiographic examination confirmed the absence of periradicular radiolucency (Figure 5q–t).

## 3. Discussion

The conditions of all three cases before and after surgical treatments are summarized in Table 1. As molars have more complex root configurations and normally undergo much higher concentrated stresses during the mastication process than anterior teeth and premolars, the VRF of endodontically treated molars has always been considered an incurable condition. Consequently, no VRF repairing treatment for molars is reported so far. This case report involved three molars representative of different locations (maxillary and mandibular) and root configurations (separate and fused), and two typical fracture directions (mesiodistal in Cases 1 &2, buccolingual in Case 3). In addition, these molars had all undergone root canal treatment followed by crown restoration, which aligns with the often-seen clinical scenarios of VRF occurrence.

The key point for repairing the VRF of molars is to firmly fix the fractures to minimize the possibility of fracture recurrence during the post-treatment mastication process. Conventional VRF repairing methods only use resin or cement to fill the fracture [23], which may offer very limited mechanical stability for fractures. This report introduced trapezoidal retention forms during the fracture line preparation, which could provide a mechanical interlocking effect and increase the resin-to-dentin bonding area to enhance the fixation stability and strength. Furthermore, this modification could probably help distribute stresses, thereby reducing the risk of stress concentration and fracture recurrence. These merits seemed supported by the outcomes of this case series. Meanwhile, the dual-layered design placed the bioceramic cement on top of the resin surface, thus promoting periodontal tissue healing. The release of ions from bioceramic cement, such as calcium (Ca^2+^) and silicate (SiO_4_^4−^), could induce the regeneration of periodontal hard tissues, which helps achieve biological sealing of the fracture zone [24]. This synergy between mechanical fixation and biological healing may create a favorable environment for the long-term success and integration of the replanted tooth.

A critical but previously overlooked factor in VRF prognosis is bacterial invasion. The fracture line creates a communication channel between the oral cavity and periapical tissues. Microbial invasion and biofilm accumulation would directly trigger a persistent inflammatory response in the adjacent periodontal tissues, initiating a pathological cascde that undermines periodontal and periapical tissue integrity [25]. In contrast to the conventional single-material repairing method, our modified dual-layered approach integrates three aspects of bacterial control. First, mechanical enlargement and preparation of the fracture line minimized the infected dentin tissues along the fracture line. Second, the resin layer provided a tight seal that blocks bacteria from re-entering the fracture site or dentinal tubules. Third, the bioceramic layer maintained a local alkaline condition to enable antimicrobial protection [26].

VRFs are often associated with severe periodontal destruction, making their healing significantly more challenging than that of isolated periapical lesions. Although the dual-layered repairing approach achieved root fracture fixation and sealing as well as a significantly reduced periodontal probing depth, the probing depth did not fully return to its optimal range (≤3 mm) during the post-operative follow-up period. In this report, mechanical barriers such as membranes were not used to cover the osseous defect. As a result, in the post-operative osseous defect area, the oral epithelium and gingival connective tissue with a faster proliferation rate than hard tissue were prone to grow into the defect [27]. This further led to insufficient bone regeneration and incomplete reconstruction of the periodontal attachment [28], which may explain the non-ideal periodontal recovery during the follow-up in this report. This problem could possibly be resolved through the combination of guided tissue regeneration (GTR) techniques [29].

While the novel dual-layered repairing method showed satisfactory treatment results, its feasibility due to the high level of surgical expertise required necessitates specialized training and experience. There are several notable limitations that leave room for improvement and further investigation. First, all three reported cases involved single-surface VRFs, and the 1.5–2 mm preparation depth was sufficient to fully encapsulate and seal the entire visible fracture line. For fractures with a depth exceeding 1.5–2 mm, the preparation depth should be adjusted to match the actual extent of the crack, ensuring that the entire visible fracture line is fully exposed and debrided. Furthermore, for VRFs involving multiple root surfaces, the current approach may not be applicable as multi-surface fractures would compromise the mechanical stability of the root even after repair. Second, the longer-term efficacy and durability of the treatment remain to be confirmed. Future studies should include more VRF cases with varying fracture types, and randomized controlled investigations need to be carried out. Additionally, further exploration is needed to determine how to combine this repairing approach with other new materials (e.g., high-strength bioceramics) and treatment techniques (e.g., GTR) to improve its treatment results for cases with severe bone defects or complex fracture patterns.

## 4. Conclusions

This case series report demonstrated for the first time that the VRF of endodontically treated molars could be successfully managed by the dual-layered repairing approach with modified fracture lines and retention forms through intentional replantation. Despite this, rigorous randomized controlled investigations with larger sample sizes and longer follow-up periods are required to verify the longer-term efficacy and durability of this approach.

## Figures and Tables

**Table 1:** Clinical and demographic features of three cases.

Variable		Case 1	Case 2	Case 3
Age (y)		30	33	31
Sex		Female	Male	Female
Tooth No.		#46	#16	#37
Fracture line direction		mesio-distal	mesio-distal	bucco-lingual
Clinical symptoms and signs	Sinus tract	√	√	×
	Deep isolated pocket detected	√	√	×
	Periodontal probing depths (mm)	263 232	438 322	333 334
	Percussion pain	√	√	√
Periradicular radiolucency		√	√	√
Extra-oral time (minutes)		14	13	11
Follow-up (months)		36	21	18
Treatment outcomes	Clinical Symptoms	Asymptomatic	Asymptomatic	Asymptomatic
	Periodontal Probing Depths (mm)	243 233	334 323	323 334
	Tooth mobility	Normal	Normal	Normal
	Radiographic Findings	Reduced radiolucency, partial bone regeneration	Reduced radiolucency, partial bone regeneration	Complete periradicular healing

#: Tooth number (FDI notation); √: Presence of the symptom/sign; ×: Absence of the symptom/sign.

## Data Availability

All data generated in this case report are included in this published manuscript.

## Abbreviations

VRF	Vertical Root Fracture
CBCT	Cone Beam Computed Tomography
RCT	Root Canal Therapy
GTR	Guided Tissue Regeneration

## References

[1] Patel S., Teng P.H., Liao W.C., Davis M.C., Fidler A., Haupt F., Fabiani C., Zapata R.O., Bose R. (2025). Position statement on longitudinal cracks and fractures of teeth. Int. Endod. J..

[2] Walton R.E. (2017). Vertical root fracture: Factors related to identification. J. Am. Dent. Assoc..

[3] Liao W.C., Chen C.H., Pan Y.H., Chang M.C., Jeng J.H. (2021). Vertical Root Fracture in Non-Endodontically and Endodontically Treated Teeth: Current Understanding and Future Challenge. J. Pers. Med..

[4] Floratos S.G., Kratchman S.I. (2012). Surgical management of vertical root fractures for posterior teeth: Report of four cases. J. Endod..

[5] Barkhordar R.A. (1991). Treatment of vertical root fracture: A case report. Quintessence Int..

[6] Dederich D.N. (1999). CO_2_ laser fusion of a vertical root fracture. J. Am. Dent. Assoc..

[7] Wang Y.L., Lee B.S., Tseng C.L., Lin F.H., Lin C.P. (2008). In vitro study of root fracture treated by CO_2_ laser and DP-bioactive glass paste. J. Formos. Med. Assoc..

[8] Moradi Majd N., Akhtari F., Araghi S., Homayouni H. (2012). Treatment of a vertical root fracture using dual-curing resin cement: A case report. Case Rep. Dent..

[9] Okaguchi M., Kuo T., Ho Y.C. (2019). Successful treatment of vertical root fracture through intentional replantation and root fragment bonding with 4-META/MMA-TBB resin. J. Formos. Med. Assoc..

[10] Hadrossek P.H., Dammaschke T. (2014). New treatment option for an incomplete vertical root fracture–A preliminary case report. Head Face Med..

[11] von Arx T., Bosshardt D. (2017). Vertical root fractures of endodontically treated posterior teeth: A histologic analysis with clinical and radiographic correlates. Swiss Dent. J..

[12] Aslan T., Esim E., Üstün Y. (2024). Finite element evaluation of dentin stress changes following different endodontic surgical approaches. Odontology.

[13] Szabó V.T., Szabó B., Paczona B., Mészáros C., Braunitzer G., Balázs Szabó P., Garoushi S., Fráter M. (2022). The biomechanical effect of root amputation and degree of furcation involvement on intracoronally splinted upper molar teeth—An in vitro study. J. Mech. Behav. Biomed. Mater..

[14] Sun Q., Han F., Fan W. (2024). A novel surgical treatment approach for the vertical root fracture of posterior teeth: A case report with 24-month review. BMC Oral Health.

[15] Zhong X., Yan P., Fan W. (2022). New approach for the treatment of vertical root fracture of teeth: A case report and review of literature. World J. Clin. Cases.

[16] Yan H., Xu N., Wang H., Yu Q. (2019). Intentional Replantation with a 2-segment Restoration Method to Treat Severe Palatogingival Grooves in the Maxillary Lateral Incisor: A Report of 3 Cases. J. Endod..

[17] De-Deus G., Canabarro A., Alves G.G., Marins J.R., Linhares A.B., Granjeiro J.M. (2012). Cytocompatibility of the ready-to-use bioceramic putty repair cement iRoot BP Plus with primary human osteoblasts. Int. Endod. J..

[18] Hursh K.A., Kirkpatrick T.C., Cardon J.W., Brewster J.A., Black S.W., Himel V.T., Sabey K.A. (2019). Shear Bond Comparison between 4 Bioceramic Materials and Dual-cure Composite Resin. J. Endod..

[19] Becker B.D. (2018). Intentional Replantation Techniques: A Critical Review. J. Endod..

[20] Lin Z., Huang D., Huang S., Chen Z., Yu Q., Hou B., Qiu L., Chen W., Li J., Wang X. (2025). Expert consensus on intentional tooth replantation. Int. J. Oral Sci..

[21] Plotino G., Abella Sans F., Duggal M.S., Grande N.M., Krastl G., Nagendrababu V., Gambarini G. (2022). Present status and future directions: Surgical extrusion, intentional replantation and tooth autotransplantation. Int. Endod. J..

[22] Nagendrababu V., Chong B.S., McCabe P., Shah P.K., Priya E., Jayaraman J., Pulikkotil S.J., Dummer P.M.H. (2020). PRICE 2020 guidelines for reporting case reports in Endodontics: Explanation and elaboration. Int. Endod. J..

[23] da Silva E.J.N.L., Dos Santos G.R., Krebs R.L., de Souza Coutinho-Filho T. (2012). Surgical Alternative for Treatment of Vertical Root fracture: A Case Report. Iran. Endod. J..

[24] Lee G.W., Yoon J.H., Jang J.H., Chang H.S., Hwang Y.C., Hwang I.N., Oh W.M., Lee B.N. (2019). Effects of newly-developed retrograde filling material on osteoblastic differentiation in vitro. Dent. Mater. J..

[25] Giardino L., Grande N.M., Savadori P., Fabbro M.D., Plotino G. (2019). Clinical and Histological Findings of Post-Treatment Infection in the Presence of Vertical Root Fracture and Apical Periodontitis: Case Reports. Eur. Endod. J..

[26] Seron M.A., Nunes G.P., Ferrisse T.M., Strazzi-Sahyon H.B., Dos Santos P.H., Gomes-Filho J.E., Cintra L.T.A., Sivieri-Araujo G. (2024). Influence of bioceramic sealers on dentinal tubule penetration and antimicrobial effectiveness: A systematic review and meta-analysis of in vitro studies. Odontology.

[27] Wang H.L., Modarressi M., Fu J.H. (2012). Utilizing collagen membranes for guided tissue regeneration-based root coverage. Periodontology 2000.

[28] Bee S.L., Hamid Z.A.A. (2022). Asymmetric resorbable-based dental barrier membrane for periodontal guided tissue regeneration and guided bone regeneration: A review. J. Biomed. Mater. Res. B Appl. Biomater..

[29] Sugaya T., Kawanami M., Noguchi H., Kato H., Masaka N. (2001). Periodontal healing after bonding treatment of vertical root fracture. Dent. Traumatol..

